# Rhinovirus Genome Variation during Chronic Upper and Lower Respiratory Tract Infections

**DOI:** 10.1371/journal.pone.0021163

**Published:** 2011-06-21

**Authors:** Caroline Tapparel, Samuel Cordey, Thomas Junier, Laurent Farinelli, Sandra Van Belle, Paola M. Soccal, John-David Aubert, Evgeny Zdobnov, Laurent Kaiser

**Affiliations:** 1 Laboratory of Virology, Division of Infectious Diseases and Division of Laboratory Medicine, University of Geneva Hospitals, Geneva, Switzerland; 2 University of Geneva Medical School, Geneva, Switzerland; 3 Department of Genetic Medicine and Development, University of Geneva Medical School, Geneva, Switzerland; 4 Swiss Institute of Bioinformatics, Geneva, Switzerland; 5 Fasteris SA, Plan-les-Ouates, Switzerland; 6 Division of Pulmonary Medicine and Clinic of Thoracic Surgery, University of Geneva Hospitals, Geneva, Switzerland; 7 Division of Pulmonary Medicine, University of Lausanne Hospitals, Lausanne, Switzerland; Institut Pasteur, France

## Abstract

Routine screening of lung transplant recipients and hospital patients for respiratory virus infections allowed to identify human rhinovirus (HRV) in the upper and lower respiratory tracts, including immunocompromised hosts chronically infected with the same strain over weeks or months. Phylogenetic analysis of 144 HRV-positive samples showed no apparent correlation between a given viral genotype or species and their ability to invade the lower respiratory tract or lead to protracted infection. By contrast, protracted infections were found almost exclusively in immunocompromised patients, thus suggesting that host factors rather than the virus genotype modulate disease outcome, in particular the immune response. Complete genome sequencing of five chronic cases to study rhinovirus genome adaptation showed that the calculated mutation frequency was in the range observed during acute human infections. Analysis of mutation hot spot regions between specimens collected at different times or in different body sites revealed that non-synonymous changes were mostly concentrated in the viral capsid genes VP1, VP2 and VP3, independent of the HRV type. In an immunosuppressed lung transplant recipient infected with the same HRV strain for more than two years, both classical and ultra-deep sequencing of samples collected at different time points in the upper and lower respiratory tracts showed that these virus populations were phylogenetically indistinguishable over the course of infection, except for the last month. Specific signatures were found in the last two lower respiratory tract populations, including changes in the 5′UTR polypyrimidine tract and the VP2 immunogenic site 2. These results highlight for the first time the ability of a given rhinovirus to evolve in the course of a natural infection in immunocompromised patients and complement data obtained from previous experimental inoculation studies in immunocompetent volunteers.

## Introduction

Human rhinoviruses (HRV) usually cause self-limited upper respiratory tract (URT) illness. However, they are increasingly reported to be associated with complications, such as asthma [1], [2], [3], chronic obstructive pulmonary disease (COPD) exacerbations [4], pneumonia, and bronchiolitis in young children [5]. HRVs have the ability to infect the lower respiratory tract (LRT) [6], [7] and can cause chronic infections in immunocompromised hosts [8], [9]. Unlike enteroviruses, most rhinoviruses replicate optimally at lower airway temperatures, which is thought to explain their URT tropism [10]. Rhinovirus strains infecting the LRT may require specific adaptative genomic changes that have not yet been identified.

For most viral infections, disease severity is the result of a close interplay between viral and host factors. The host immune status is known to play a critical role for viral clearance and resolution of infection. Abnormal innate immune responses to HRV have been observed in individuals presenting HRV-induced COPD or asthma exacerbation [11], [12]. By contrast, reports are rare on the implication of viral determinants in HRV disease severity. Palmemberg et al proposed that the rhinovirus 5′UTR polypyrimidine tract may affect virulence [13]. At the serotype or species level, a clear association between the genotype and induced pathology remains to be demonstrated. HRVs were previously classified into two species, HRV-A and HRV-B. In 2006, a third species, HRV-C, was identified [14], [15], [16], [17], [18], [19] and appears to be highly prevalent and circulating worldwide [20], [21], [22]. Several studies have suggested that HRV-C types are prone to induce more severe illness in children [17], [21], [23], [24], [25].

Due to their error-prone RNA polymerase, RNA viruses are subject to constant evolution. The error rate of picornavirus RNA polymerases has been estimated to range between 10^−3^ and 10^−4^ errors/nucleotide/cycle of replication [26], [27]. This variability is a driving force for virus evolution. The number of genera, species, and serotypes illustrates this diversity (http://www.picornaviridae.com) with at least 74 HRV-A and 25 HRV-B serotypes identified, and 61 HRV-C types proposed [22].

While the evolution of rhinoviruses has been well characterized at the serotype or species level [13], [28], [29], [30], [31], [32], little is known about the diversity generated during the course of a natural human infection. Several studies have investigated the genomic variability of enteroviruses and vaccine-derived polioviruses during chronic infections in immunocompromised patients, but there is no such report for rhinoviruses [33], [34], [35], [36].

Our group analyzed recently HRV-39 genome evolution in experimental immunocompetent human infections [37]. We estimated an *in vivo* mutation frequency of 3.4×10^−4^ mutations/nucleotides over the entire open reading frame (ORF) during a five-day acute infection period, and identified regions of mutation hot spots in the viral capsid (VP1, VP2 and VP3), and 2C and 3C genes.

In the present study, we performed first a phylogenetic analysis to compare the relative distribution of HRV species or serotypes according to the respiratory site (URT versus LRT) and in protracted infection in hospital patients and lung transplant recipients. In the latter group, we evaluated the frequency of mutations and characterized mutation hot spot regions during the course of five natural human protracted infections, each caused by a different rhinovirus serotype. In one case, we followed intra-host rhinovirus genome variation at different time points in the URT and LRT over a period of 27 months using both classical and ultra-deep sequencing methods.

## Materials and Methods

### Ethics statement

Written informed consent was obtained from all individuals prior to study participation. The studies were approved by the Institutional Review Board of the University of Geneva Hospitals.

### RNA extraction, RT and real-time PCR assay

Clinical specimens were extracted by the HCV Amplicor Specimen Preparation kit (Roche, Rotkreuz, Switzerland), TRIzol (Invitrogen, Carlsbad, CA, USA), or Easymag (bioMérieux, Geneva, Switzerland), according to the manufacturers' instructions. Reverse transcription (RT) and real-time PCR with the “Panenterhino/Ge/08” primers and probe assay were performed as previously described [38]. Picornavirus-positive samples were detected in patients enrolled in a cohort of lung transplant recipients (September 2008 to November 2010) [6], [39] and in hospital patients screened by the routine laboratory of the University of Geneva Hospitals (February to November 2009) (Table S1).

### Sequencing

Complete genome sequences were obtained from the LRT and URT samples of five chronically-infected individuals collected at different time points during the course of infection. Fragments were amplified by PCR using degenerate primers designed to anneal highly-conserved sequences of the corresponding HRV reference strain. Overlapping fragments were assembled and, when necessary, specific non-degenerate primers were designed to fill the gaps between the original PCR products. All primers used in this study are listed in Table S2. PCR (primers 46 and P1.204) and semi-nested PCR (primers 47 and P1.204, Table S2) were performed for VP4/VP2 sequences.

PCR products were purified with the microcon columns (Millipore, Zug, Switzerland) before sequencing. Each PCR product was sequenced at least twice. Nucleotide changes were confirmed by a second PCR and sequencing. Chromatograms produced with the ABI Prism 3130XL DNA Sequencer (Applied Biosystems, PE Europe BV, Basel, Switzerland) were directly imported for proofreading with the Geneious Pro 5.0.3 software (Biomatters Ltd., Auckland, New Zealand). All sequences are available at GenBank under accession numbers HM347236-727; JF285163-176; JF285179; JF285186; JF285192,193,195,197,198,200,204,206-8,210,213-214,224-225, JF285228-307.

### Phylogenetic analysis

Alignments were constructed using Muscle [40] with a maximum of 64 iterations. Multiple FastA was converted into PHYLIP format (for tree-building) with the EMBOSS program ‘seqret’ [41]. Trees were built with PhyML [42] using the GTR model BIONJ for the initial tree and optimized tree topology and branch lengths. Trees with fewer than 50 species used 16 rate categories and larger trees used 8. Transition/transversion ratio, proportion of invariant sites, and the shape parameter (alpha) of the gamma distribution were estimated. Tree processing (including rooting, computation of support values, and displaying) was done with the Newick Utilities [43].

### Statistical tests

All statistical data analyses were carried out using the SAS/STAT software version 9.1.3 (SAS Institute Inc., Cary, NC, USA). Associations between HRV species, infection site, and study cohorts were evaluated using chi-square tests when possible, or Fisher's exact tests when some expected values were smaller than 5.

### Full-length genome amplification for ultra-deep sequencing

URT and LRT full-length sequences were obtained by amplification of eight overlapping nested PCR products with forward and reverse primers (Table S2). RT-PCR assays were performed in duplicate to discard any mutations introduced by polymerase errors. We used Platinium Taq DNA Polymerase High Fidelity (Invitrogen) for PCRs, according to the manufacturer's instructions. All amplicons were purified with the microcon columns (Millipore) before sequencing. The eight overlapping PCRs were pooled at equimolar concentrations and used for ultra-deep sequencing for each individual sample.

### Ultra-deep sequencing analysis

#### Samples

Libraries were prepared according to the manufacturer's protocol (Illumina, Inc., San Diego, CA, USA) using indexed adapters designed by Fasteris (Fasteris SA, Plan-les-Ouates, Switzerland) (see Methods S1).

#### Genome analyzer run

Libraries were pooled and sequenced on an Illumina Genome Analyzer GAIIx (Illumina) single-read channel for 76 cycles using a version 4 sequencing kit. We performed base-calling using Illumina pipeline RTA SCS.2.6 and CASAVA 1.6, which produced over 24 million pass filter reads attributed unambiguously or 1.85G bases.

#### Bioinformatics data analyses

Mapping was performed using the MAQ software version 0.7.1 (http://maq.sourceforge.net/maq-man.shtml). An average of 95% reads were mapped on the “F78 URT 1.4 m” consensus sequence for each sample analyzed with a mean coverage of 21.2K reads per nucleotide. Mapping results were used to extract a list of single nucleotide polymorphisms (SNPs) throughout the genome (see Supplementary method: bioinformatics data analyses).

#### Mutation analysis along the whole HRV genome

Density of mutations was represented with a Gaussian kernel density function using a smoothing band width of 0.1 kernel standard deviation. Curves are shown at the same scale and normalized so that a value of 1 is the highest density found over all genomes. Graphs, including kernel estimates, were produced with the R statistical package.

#### HRV ORF amino acid conservation plot

The conservation plot was computed with the EMBOSS package Plotcon program using default parameters, except for the sliding window size, which was set to 30 aa. The same program was used in text mode to compute the mean and standard deviations.

## Results

### Prevalence of each rhinovirus species in protracted and LRT infections

We reported previously three cases of patients chronically infected with a unique rhinovirus strain [8]. Since then, we have identified two additional similar cases. All five patients were immunocompromised lung transplant recipients infected both in the URT and LRT (Table 1). Three were infected with a HRV-A species (HRV-A64, -A24 and -A9), and two with a HRV-B species (HRV-B3 and -B27).

**Table 1:** Baseline and main characteristics of lung transplant recipients with chronic rhinoviral infection.

Patient§	Year of birth	Date of first HRV positivity	Outcome after first HRV positivity	Respiratory site infected^1^	Nearest rhinovirus genotype	Time of complete genome sequence afterfirst HRV positivity		Mutations/nt/day between first and last complete genome sequence
		Duration of infection (months)				URT^1^	LRT^1^	
P37^2^	1937	24-Jan-01	Died after 13 m	URT and LRT	HRV-A64	*0 d	-	3.88×10^−05^
						*15 d	-	
		11 m				*86 d (2.9 m)	-	
						-	*229 d (7.6 m)	
V38^2^	1938	15-Oct-04	Recovered	URT and LRT	HRV-B3	76 d (2.5 m)	-	9.54×10^−06^
		8 m				188 d (6.3 m)	-	
						208 d (6.9 m)	-	
A46^2^	1946	18-Dec-03	Died after 18 m	URT and LRT	HRV-B27	201 d (6.7 m)	201 d (6.7 m)	NC
		15 m						
R58	1958	13-Jan-04	Recovered	URT and LRT	HRV-A24	7 d	7 d	2.34×10^−05^
		3.3 m				23 d	-	
						86 d (2.9 m)	-	
F78	1978	4-Jul-06	Died after 27.7 m	URT and LRT	HRV-A9	42 d (1.4 m)	42 d (1.4 m)	7.27×10^−06^
						-	217 d (7.2 m)	
						287 d (9.6 m)	-	
						-	462 d (15.4 m)	
		27.4 m				-	506 d (16.9 m)	
						731 d (24.4 m)	731 d (24.4 m)	
						798 d (26.6 m)	798 d (26.6 m)	
						821 d (27.4 m)	821 d (27.4 m)	

1 Upper respiratory tract specimens (URT) are nasopharyngeal swabs or aspirates; lower respiratory tract specimens (LRT) are bronchoalveolar lavage fluids.

2 Partial VP1 sequence of these cases has been reported previously [8].

* Sequence obtained from cell culture isolates.

-: not available; NC: not calculated in the absence of two time points; d: day; m: months.

§ Patients are designated by the first letter of their family name and the last two numbers of their birth year.

**Figure 1:**
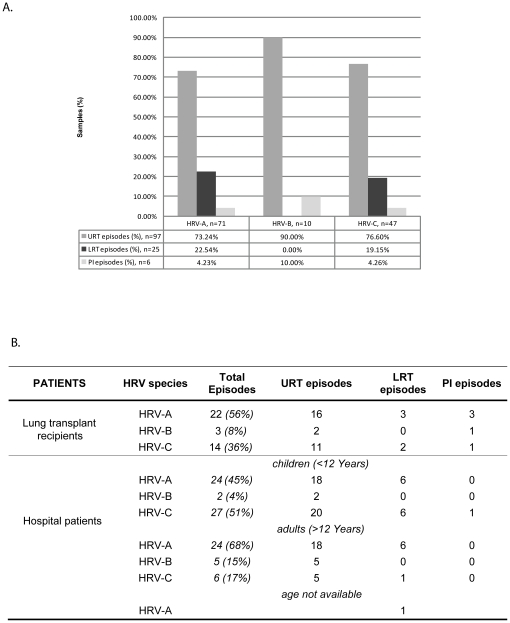
Repartition of upper respiratory tract, lower respiratory tract, and protracted infection episodes according to HRV species. A. Percentage of each species associated with upper respiratory tract infection (URT), lower respiratory tract infection (LRT), and protracted infection (PI) among lung transplant recipients and hospital patients. B. Detailed repartition of URT, LRT and PI episodes in lung transplant recipients, and pediatric and adult hospital patients according to HRV species. Details of HRV-A, -B and -C types are shown in Figures S1A to C.

We genotyped strains found in nasopharyngeal specimens (URT), bronchoalveolar lavage fluids (LRT), and strains identified in cases with infections lasting more than three weeks (protracted infection) to assess whether members of the HRV-A, -B and -C species were equally represented in the LRT and/or in the case of protracted infection. Samples were collected from a prospective cohort of lung transplant recipients screened routinely from September 2008 to November 2010, as well as hospital patients screened at the routine laboratory of the University of Geneva Hospitals between February and November 2009 (Table S1). VP4/VP2 sequencing was performed on all positive cases whenever possible. Sequences were obtained for 50 HRV-positive specimens collected from the lung transplant recipient cohort, and 94 specimens collected from hospital patients (56 from children, 36 from adults, and 1 with age not available). Phylogenetic analysis was performed on all sequences obtained, as well as corresponding sequences of HRV-A and -B reference types and proposed HRV-C reference types [22]. Of the 144 sequenced samples, 127 represented different infectious episodes with 71 (55.5%) due to HRV-A types, 10 (7.8%) to HRV-B types, and 47 (36.7%) to HRV-C types (Figure 1 and Figures S1A, B, and C).

No association was observed between a given rhinovirus species and the type of infection (URT, LRT, protracted infection) in the whole study population (Fisher's exact test, 4DF; P = 0.41). HRV-A and -C were present at similar frequencies among LRT infections (22.45% and 19.15%, respectively) in lung transplant recipients and hospital patients (Figure 1). We found also a good representation of different rhinovirus serotypes in all types of specimens (URT, LRT, protracted infection) (Figure S1). By contrast, there was a clear association between the populations studied (lung transplant recipients versus adult and pediatric hospital patients) and the type of infection (URT, LRT, protracted infection) (Fisher's exact test, 2DF; P<0.05). Six protracted infections were observed; five in lung transplant recipients and one in a 3-month-old infant. Finally, we observed an association between the infecting HRV species and patient age (Fisher's exact test, 2DF; P<0.05). The proportion of HRV-C positive samples (LRT, URT, protracted infection) was significantly higher in pediatric hospital patients than in adult hospital patients (51% versus 17%, respectively) (chi-Square 1DF; P<0.05) (Figure 1B).

### Mutation frequency and mutation hot spots in chronic cases

**Figure 2:**
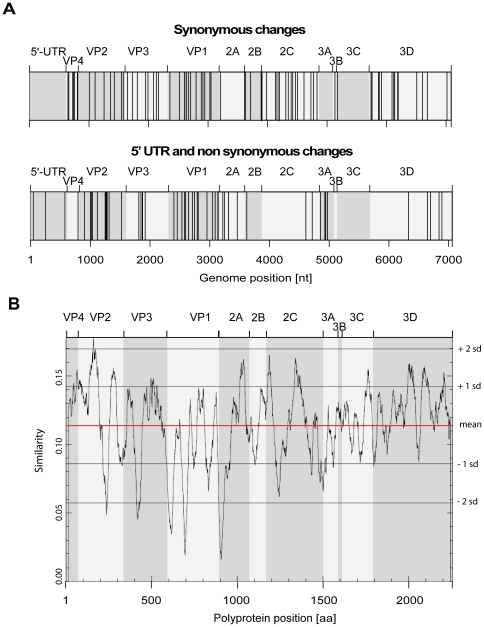
Synonymous and non-synonymous mutation hot spot regions. A. The synonymous (upper panel), 5′UTR and non-synonymous (lower panel) changes observed between the first and last samples sequenced from patients P37, V38, R58 and F78, as well as between the URT and LRT samples from patient A46, were plotted together along the HRV-2 genome (GenBank accession number X02316) to map mutation hot and cold spot regions. B. The conservation plot was calculated based on an alignment of the amino acid sequence of 99 rhinovirus serotypes and clinical strains as previously published [13]. The average sequence similarity measure for the ORF was 0.114 (red line). sd: standard deviation.

Complete genome sequences were obtained at different time points in the URT and/or LRT for the five chronically-infected lung transplant recipients (Table 1). The number of substitutions per nucleotide and per day is reported in Table 1 for each patient, except for A46 for whom samples collected at different time points were not available. Calculated mutation frequencies ranged between 7.27×10^−6^ and 3.88×10^−5^. No overlapping mutations in the URT or LRT were found between the five patients. However, superimposition of all changes along a reference genome (HRV-A2) (Figure 2A) revealed that whereas synonymous changes were distributed along the genome without any apparent specific pattern, non-synonymous mutations were clustering mostly in the capsid genes VP2, VP3 and VP1, the first half of 2A genes, and in 3A genes. Cold spot regions were located in the viral capsid gene VP4, as well as in 2B, 2C, 3B, 3C, and most of 3D. Only three changes were found in the 5′UTR and one in the 3′UTR. The variation hot spots observed in the capsid genes were similar to the amino acid variability observed among HRV reference types (Figure 2B).

### Intra-host rhinovirus genome variation according to time and respiratory site

**Figure 3:**
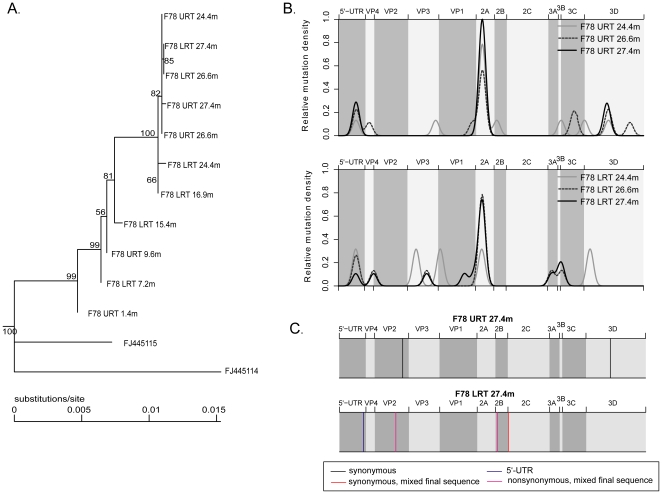
Intra-host genetic variation in patient F78 at the majority and minority population level. A. Phylogenetic tree of the 11 complete genome sequences corresponding to samples collected over a 27.4-month infection period in the URT and LRT of patient F78, as well as HRV-A67 reference type (FJ445149) and 3 HRV-9 complete genome sequences (FJ445114,15,17). HRV-A 67 and FJ445117 were used as outgroups to root the tree and are not shown. B. Minority mutation repartition along the HRV genome as recorded for the last 3 URT and LRT samples collected from patient F78. Curves indicate minority mutation densities (estimated by a Gaussian kernel function) along the genome. C. Changes specific to the URT and LRT. The sequences of the last URT and LRT samples collected from patient F78 were compared to the first complete genome sequence (F78 URT 1.4 m) and changes specific to the URT (upper panel) and LRT (lower panel) are mapped along the HRV genome.

Genetic variation was extensively studied for patient F78 as several samples were available throughout his entire 27-month infection period, including samples collected the same day in the URT and LRT. A phylogenetic tree (Figure 3A) performed with 11 complete genome sequences showed that the URT and LRT viral populations did not segregate for at least 26 months. Furthermore, a LRT subpopulation present in a sample collected after 15.4 months was the founder of all URT and LRT viral populations later collected. The non-synonymous changes that appeared and subsequently fixed were located in VP2 (3), VP3 (1), VP1 (6), 2C (1), 3A (1), and 3D (1). The mutation rate then slowed down shortly before the patient's death (4.3×10^7^ and 1.3×10^8^ viral copy/ml for the last URT and LRT, respectively), despite a high viral load at the end of our follow-up period. This may account for the overall slower mutation rate calculated for this patient when compared with those infected for a shorter period (Table 1). The last three samples collected simultaneously in both the URT and LRT were further analyzed by high throughput sequencing to compare variation at the minority population level (≥5% of the total population). Most minority variants present in the LRT after 26.6 months persisted in this site, but were absent in the corresponding URT viral populations (Figure 3B). A similar observation was made with the majority variants and demonstrates that the genomes of the URT and LRT viral populations varied separately, despite high replication levels at both sites. Of note, several samples from this patient (including sample F78 LRT 26.6 m) were successfully grown in cell culture. Finally, the sequences of the last URT (F78 URT 27.4 m) and LRT (F78 LRT 27.4 m) samples were compared to the first complete genome sequence (F78 URT 1.4 m) and changes specific to the URT (upper panel) and LRT (lower panel) were mapped along the HRV genome (Figure 3C). Specific synonymous changes were found in both the URT and LRT sequences. However, non-synonymous changes were observed only in the LRT where we identified one change in the 5′UTR polypyrimidine tract [44], one non-synonymous change in the VP2 immunogenic site 2, and one non-synonymous change in protein 2A [32], [45].

## Discussion

Based on our phylogenetic analysis, the respective frequency distribution of strains infecting the URT and LRT did not reveal any apparent correlation between a given HRV serotype or species and their ability to infect the LRT. In both lung transplant recipients and hospital patients, HRV-A and -C strains presented a similar propensity to infect the LRT. No HRV-B strains were found to be associated with LRT, but the relatively small number of infected samples precludes us from drawing firm conclusions. In this respect, we have previously described chronic LRT HRV-B infections [8]. In our study, protracted infections were all found in hosts with a very low level of immunity (mostly lung transplant recipients and a 3-month-old infant). These results suggest that host factors are determinants of disease outcome, rather than virus type.

Five lung transplant recipients were chronically infected with HRV during periods of time ranging from three to 27 months, thus allowing us to study the intra-host genetic variation during natural chronic infections in immunocompromised hosts. Complete Sanger-based and ultra-deep genome sequencing were performed at different time points and sites during infection. Mutation mapping along the HRV genome pointed out that synonymous changes were roughly spread along the entire ORF, whereas non-synonymous changes clustered mostly in the capsid VP2, VP3, and VP1 genes. These capsid genes are also the most variable during acute infections in immunocompetent hosts [37], as well as the most diverse among the 99 HRV reference types [13]. The VP2, VP3 and VP1 proteins form the outer shell of the virion and are under immune pressure, which explains their fast evolution. The localization of mutation hot spots in these genes in immunocompromised hosts may appear unexpected. However, although these patients are highly immunosuppressed at the time of transplantation, immunosuppressive therapy is then adapted to prevent graft rejection and to preserve a residual cellular and humoral immunity that may account for this observation. This residual immunity is also necessary for the control and recovery of viral and bacterial infections observed in these patients.

We calculated the mutation frequency occurring during these five chronic infections, each caused by a distinct HRV serotype and for various periods of time. Mutation frequency was different in each host and likely reflects different viral replication levels, different host environments, and different durations of infection. The calculated mutation frequency ranged between 7.27×10^−06^ and 3.88×10^−05^ change/nt/day. Recent data on HRV-39 genome evolution over a 5-day acute experimental infection in human immunocompetent volunteers revealed a mutation frequency of 3.4×10^−4^ change/nt (equivalent to 6.83×10^−5^ change/nt/day) [37]. Therefore, the HRV mutation frequency observed in some immunocompromised hosts infected over months was of the same order of magnitude as that observed in acute 5-day infections.

Extensive analysis of rhinovirus genome modification was performed in the case of one patient (F78) infected for more than two years with a HRV-A9 strain. Phylogenetic analysis of 11 complete genome sequences (including ultra-deep sequences performed at three different time points) revealed that the URT and LRT populations were phylogenetically indistinguishable for a prolonged period of time, either because of co-variation or, more likely, constant viral population mixing. Once potentially adapted to the host through the accumulation of non-synonymous changes mainly in the capsid gene VP1, the viral populations appeared to evolve more slowly. Finally, we observed signatures of putative adaptation to lower airway conditions after several months of infection. Indeed, the last two LRT populations studied presented specific changes in the 5′UTR polypyrimidine tract and two non-synonymous changes, one in the VP2 immunogenic site 2 and the second in protein 2A. These changes were absent in the URT population and, upon their appearance, the two populations remained separated as confirmed by ultra-deep sequencing analysis. Although the direct impact of these changes on virus growth ability at lower airway conditions remains to be demonstrated experimentally, the very high viral load observed at this stage suggests that both populations were adapted to their sites.

Taken together, our data suggest that immunocompromised patients cannot clear viral infections as immunocompetent individuals, and represent a potential reservoir for the emergence of new variants and inter-host transmission due to longer chronic viral infection. In addition, these patients may be co-infected by two viruses, thus opening the door to recombination, another putative driving force of rhinovirus evolution [30]. With the emergence of new therapies and progress in transplantation, the population of immunocompromised patients is constantly increasing. Our results suggest that this could accelerate the ability of viruses to adapt to the host, evolve, and propagate and may favor a more rapid emergence of new viral variants.

## Supporting Information

Figure S1 **Repartition of protracted and lower respiratory tract infections among the HRV-A (panel A), HRV-B (panel B), and HRV-C (panel C) reference serotypes.** VP4-VP2 cladograms of rhinoviruses isolated from lung transplant recipients (blue) and routinely screened hospital patients (red) (Table S1), as well as the 74 HRV-A (panel A), 25 HRV-B (panel B), and 61 proposed HRV-C reference types (black) (panel C). SV1 (GenBank accession number AY064708) was used as an outgroup. LRT and PI are highlighted by arrows and black lines respectively.
(PDF)

Table S1 **Samples collected from the lung transplant recipient cohort and samples from hospital patients screened by the routine laboratory of the University Hospitals of Geneva.** *Samples are designated by the first letter of their family name followed by the last two numbers of their birth year (eg. B48), the month and year of sample collection (eg. 0209), and the site of sampling (U, upper respiratory tract; L, lower respiratory tract).
(XLS)

Table S2 **Primers used to amplify and sequence the genomes of the clinical strains described in** **Table 1** **.**
(XLS)

Methods S1 **Ultra-deep sequencing analysis.**
(DOC)
