# Supplementary figures and images for: Rhinovirus Genome Variation during Chronic Upper and Lower Respiratory Tract Infections
Source: PLoS One. 2011 Jun 21;6(6):e21163. doi: 10.1371/journal.pone.0021163 (PMC3119694; doi:10.1371/journal.pone.0021163)

A

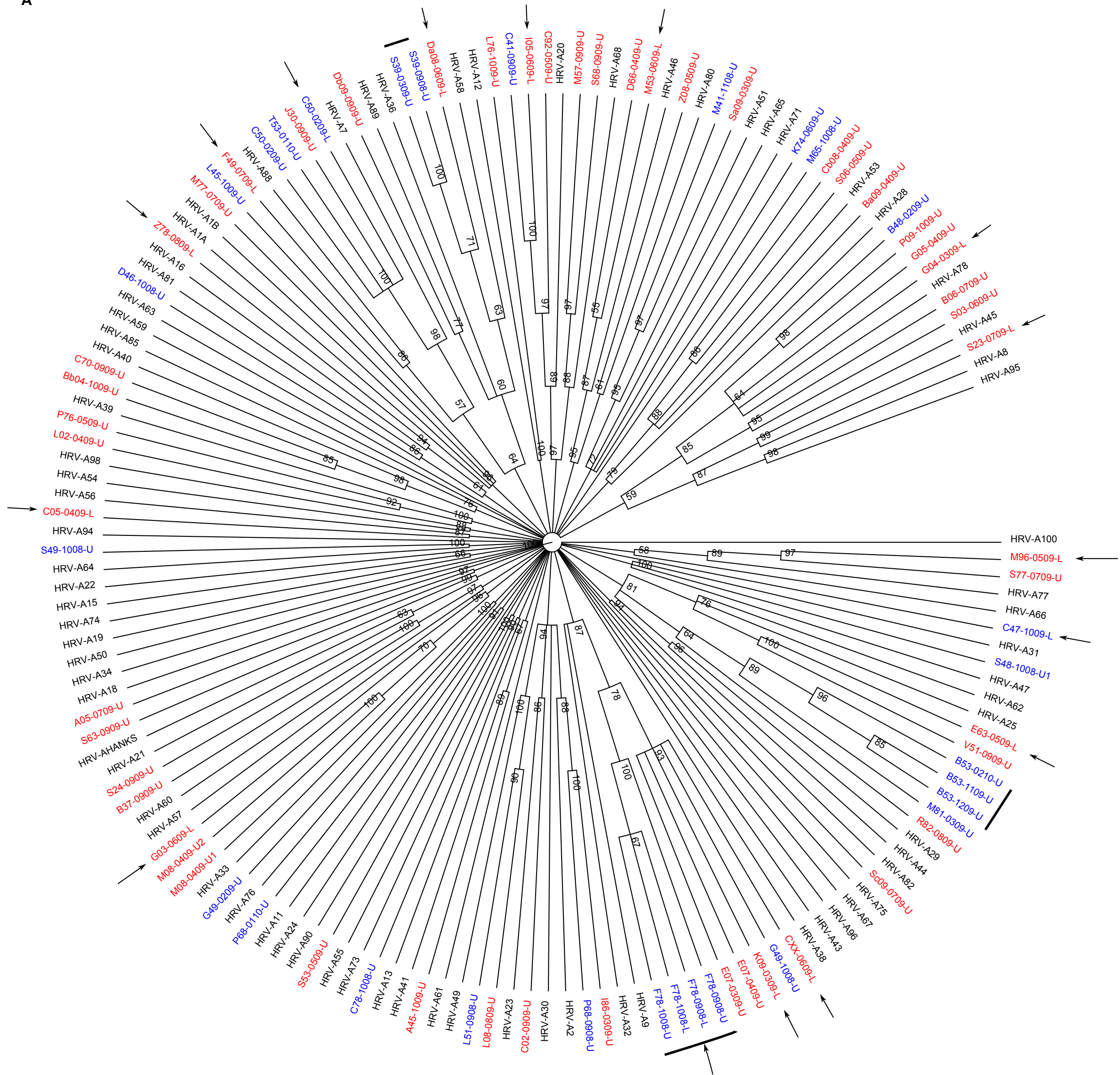

**B**

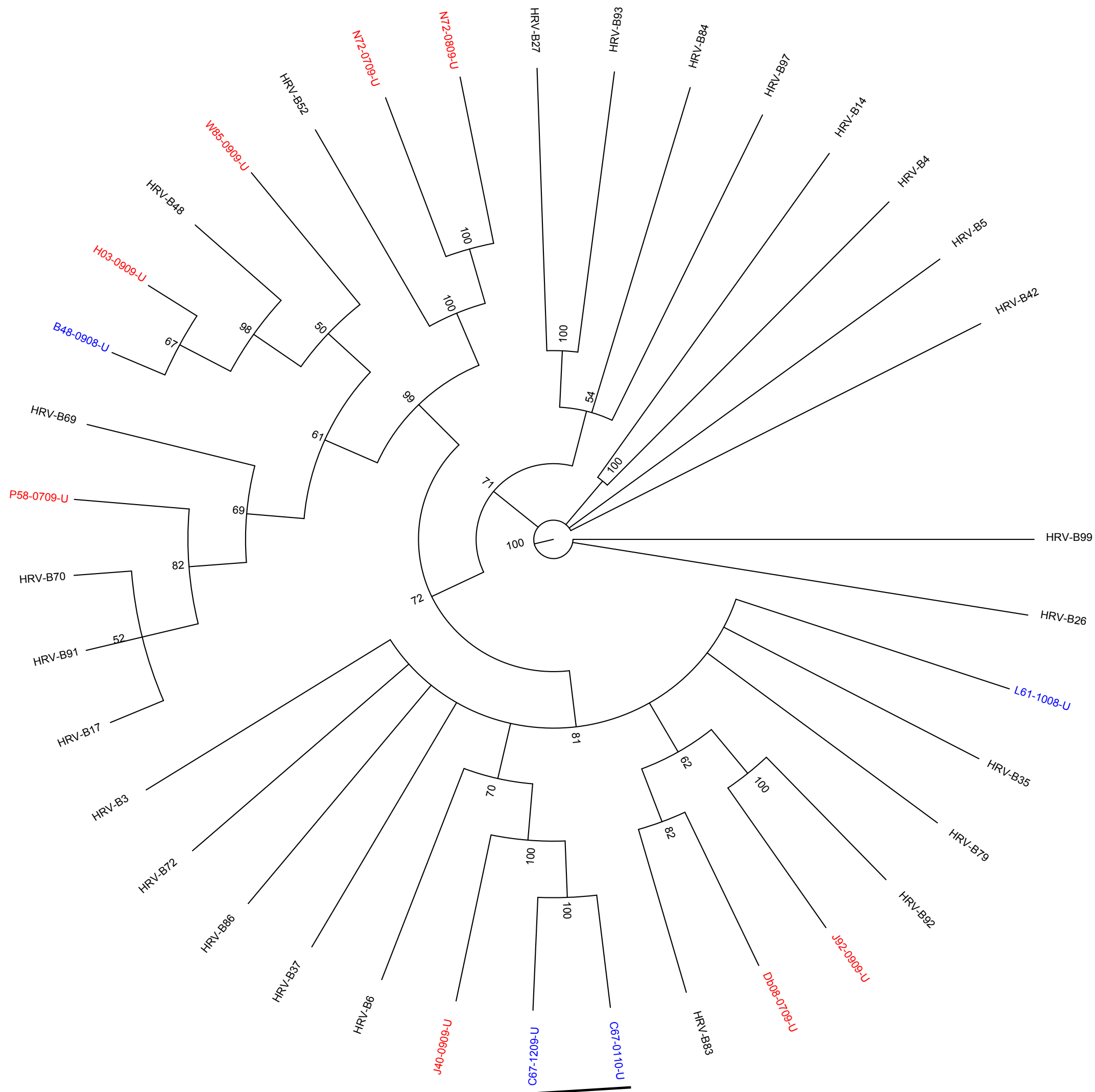

C

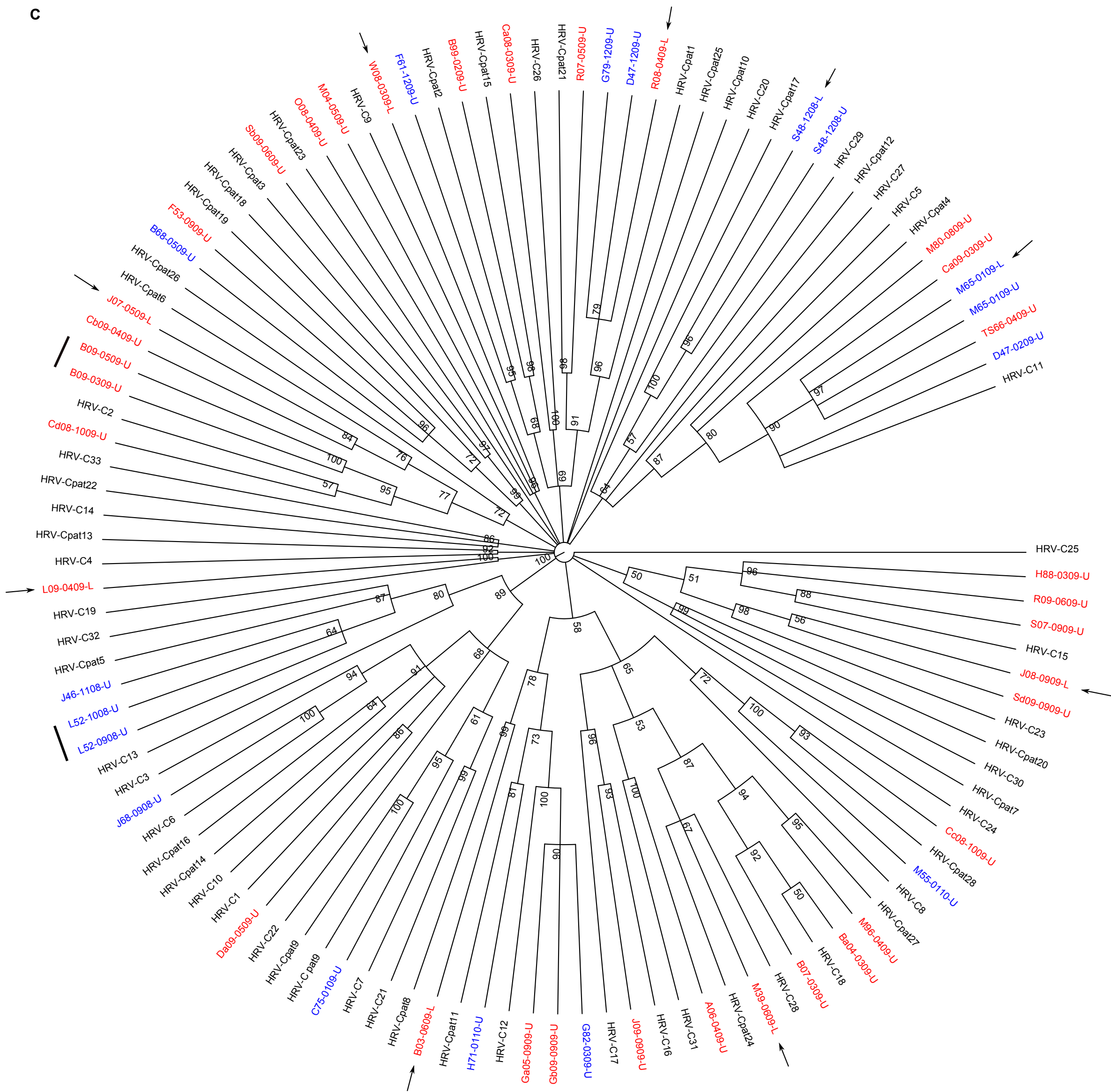

Supplement: Figure S1 — Repartition of protracted and lower respiratory tract infections among the HRV-A (panel A), HRV-B (panel B), and HRV-C (panel C) reference serotypes. VP4-VP2 cladograms of rhinoviruses isolated from lung transplant recipients (blue) and routinely screened hospital patients (red) (Table S1), as well as the 74 HRV-A (panel A), 25 HRV-B (panel B), and 61 proposed HRV-C reference types (black) (panel C). SV1 (GenBank accession number AY064708) was used as an outgroup. LRT and PI are highlighted by arrows and black lines respectively. (PDF) [file pone.0021163.s001.pdf]
