# Supplementary material for: Rhinovirus Genome Variation during Chronic Upper and Lower Respiratory Tract Infections
Source: PLoS One. 2011 Jun 21;6(6):e21163. doi: 10.1371/journal.pone.0021163 (PMC3119694; doi:10.1371/journal.pone.0021163)
Supplement: Methods S1 — Ultra-deep sequencing analysis. (DOC) [file pone.0021163.s004.doc]

**Methods S1**

**Ultra-deep sequencing analysis**

**Library preparation.** Each library consists of eight PCR products pooled at equimolar concentration and fragmented by nebulization. After end repair to generate blunt ends and the addition of one A at the 3’ends, fragments were ligated with a modified genomic adapter containing an internal 6 base index at its 3’end (the index was sequenced separately). Purification on agarose gel was used to recover fragments of approximately 300 bp. PCR amplification was performed for 15 cycles using the Phusion Polymerase (Finnzymes, Espoo, Finland). Libraries were purified and quality controlled by cloning a 1 ul aliquot into a pCR4Blunt-TOPO plasmid (Invitrogen) and capillary sequencing of eight clones to verify correct constructs and inserts. We quantified the libraries using BioAnalyzer (Agilent, Basel, Switzerland) and Q-bit (Invitrogen) and diluted to 10 nM.

**Bioinformatics data analyses.** Reads were mapped on the “F78 URT 1.4m” consensus sequence accepting a maximum of two mismatches within the first 24 bases. Reads mapping to several positions on the reference sequence with the same mapping quality (i.e. number of mismatches and quality of the bases generating the mismatches) were attributed randomly to one of the possible locations with a 0 mapping quality. The percentage of base at each position on the reference sequence was recorded. Assuming a beta distribution of the data, the upper and lower boundaries of these percentages were computed. Two bases at a given position with a lower boundary above 5% were required to select a SNP. In addition, we selected positions with one SNP base of at least 20% in regard to the reference sequence to recover unambiguous mutations that the previous filter would not have selected. Finally, the SNPs obtained were compared between the replicates for cross-validation.
